# Analysis of a conserved RGE/RGD motif in HCV E2 in mediating entry

**DOI:** 10.1186/1743-422X-6-12

**Published:** 2009-01-26

**Authors:** Katharina B Rothwangl, Lijun Rong

**Affiliations:** 1 Department of Microbiology and Immunology, University of Illinois at Chicago, Chicago, IL 60612, USA

## Abstract

**Background:**

Hepatitis C virus (HCV) encodes two transmembrane glycoproteins E1 and E2 which form a heterodimer. E1 is believed to mediate fusion while E2 has been shown to bind cellular receptors. It is clear that HCV uses a multi-receptor complex to gain entry into susceptible cells, however key elements of this complex remain elusive. In this study, the role of a highly conserved RGE/RGD motif of HCV E2 glycoprotein in viral entry was examined. The effect of each substitution mutation in this motif was tested by challenging susceptible cell lines with mutant HCV E1E2 pseudotyped viruses generated using a lentiviral system (HCVpp). In addition to assaying infectivity, producer cell expression and HCVpp incorporation of HCV E2 proteins, CD81 binding profiles, and conformation of mutants were examined.

**Results:**

Based on these characteristics, mutants either displayed wt characteristics (high infectivity [≥ 90% of wt HCVpp], CD81 binding, E1E2 expression, and incorporation into viral particles and proper conformation) or very low infectivity (≤ 20% of wt HCVpp). Only amino acid substitutions of the 3^rd ^position (D or E) resulted in wt characteristics as long as the negative charge was maintained or a neutral alanine was introduced. A change in charge to a positive lysine, disrupted HCVpp infectivity at this position.

**Conclusion:**

Although most amino acid substitutions within this conserved motif displayed greatly reduced HCVpp infectivity, they retained soluble CD81 binding, proper E2 conformation, and incorporation into HCVpp. Our results suggest that although RGE/D is a well-defined integrin binding motif, in this case the role of these three hyperconserved amino acids does not appear to be integrin binding. As the extent of conservation of this region extends well beyond these three amino acids, we speculate that this region may play an important role in the structure of HCV E2 or in mediating the interaction with other factor(s) during viral entry.

## Background

To complete its replication cycle, a virus needs to gain access to the cell cytoplasm by crossing the plasma membrane of a host cell. For enveloped viruses, such as hepatitis C virus (HCV), this entails binding at the cell surface, followed by endocytosis [1]. Entry of HCV is an intricate process that is not fully understood. Evidence indicates that HCV requires multiple receptors to invade host cells [2-4]. However, the key components that mediate susceptibility to HCV still remain elusive. It is known that the HCV E1 and E2 glycoproteins mediate the tightly regulated process of cell binding and membrane fusion.

Several cellular surface molecules have been implicated in HCV entry, including: CD81 [5-8], scavenger receptor class B type I (SR-BI) [9-12], the low-density lipoprotein receptor (LDLR) [13,14], Claudin-1, 6 and 9 [15-17], dendritic-cell-specific intercellular adhesion molecule 3-grabbing nonintegrin (DC-SIGN) [18-20] and Liver/lymph node-specific intercellular adhesion molecule-3-grabbing integrin (L-SIGN) [21,22]. While L-SIGN and DC-SIGN are not expressed on hepatocytes, it is believed that dendritic cells expressing these molecules facilitate persistent infection by capturing and delivering the virus to the liver. SR-BI is a multiligand receptor that binds several lipoproteins, including HDL, LDL and VLDL. It is primarily expressed in the liver and facilitates the uptake of lipids [23,24].

**Figure 1:**
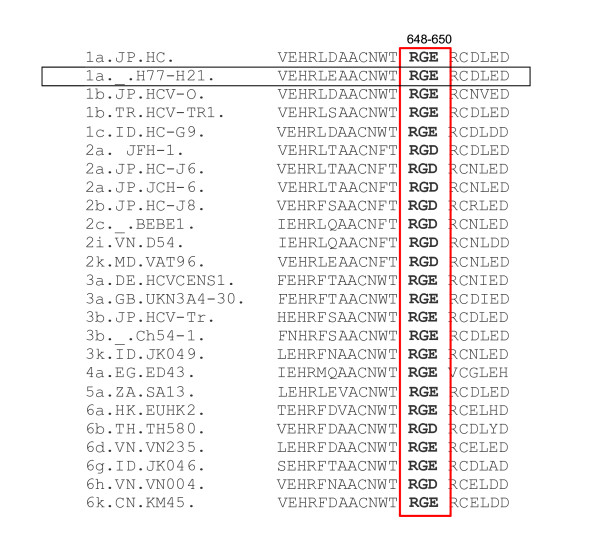
**Conserved RGE or RGD motif of hepatitis C virus E2**. HCV strains from the Los Alamos HCV sequence database were aligned. The conserved RGE or RGD motif is boxed in red. Amino acids are numbered relative to the AUG start codon of the H77 strain (boxed in black) used in this study.

As the HCV E2 glycoprotein is exposed to so much selective pressure and the high error rate inherent in RNA viruses [25], regions conserved across genotypes suggest an important role in virus viability. Sequence alignment of E2 of several genotypes of HCV revealed a highly conserved RGE or RGD motif at amino acids 648–650 (Fig. 1), which is also flanked upstream and downstream by a large stretch of sequence conservation. The presence of RGD/RGE in HCV E2 led us to speculate that integrins might be involved in HCV binding to the target cells, as RGD and RGE have been identified as an integrin binding motif [26-28].

Integrins belong to a large family of cell adhesion molecules (CAMs) [29,30]. They are responsible for cell and extracellular matrix interactions as well as cell-cell interactions. Unlike many other cell surface receptors, integrins bind with very low affinity, however they are present in much larger numbers than other receptors. The binding of integrins can be compared to Velcro®, low affinity but with many interaction sites. Integrins are heterodimeric receptors made up of an α and β subunit [31,32].

In this study, to define the role of the conserved RGE/RGD motif of E2 in HCV binding and entry, individual substitutions of these three amino acids were generated via site-directed mutagenesis. Our results suggest that although RGE/D motif of HCV E2 is critical for viral entry, integrins are probably not involved. Based on the size of this conserved region, we speculate that it is of a structural/functional nature.

## Results

### Identification of a highly conserved RGE/RGD motif

Sequence alignment of several genotypes of HCV E2 reveals all genotypes contain either a RGE or RGD motif upstream of the transmembrane domain of E2 (aa 648–650) (Fig. 1). Furthermore, a large region of sequence conservation around RGE/RGD is observed, suggesting a role(s) of this region in structure or/and function of E2.

### Effect of amino acid substitutions on the infectivity of hepatitis C virus pseudoparticles

**Figure 2:**
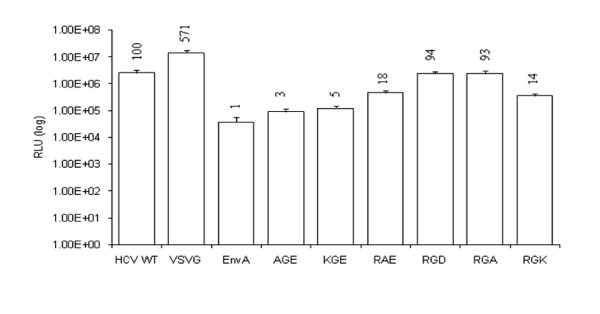
**Amino acid substitutions within the conserved RGE motif of E2 dramatically affect hepatitis C virus pseudoparticle entry**. 293 T cells were cotransfected with HIV-luc packaging vector along with HCV E1E2 mutant expression plasmids. HCVpp was harvested at 48 h PI and used to infect Huh7 cells. Infectivity was measured 72 h PI using a luciferase reporter assay. Numbers shown above the bars are infectivity of each mutant expressed as a percentage of the infectivity observed for the wild-type (wt) H77 E1E2. Values shown are the mean and standard error for a minimum of three assays.

To determine if the conserved RGE/RGD motif of HCV E2 is vital for mediating HCVpp entry, substitutions were generated within the context of H77 E2. Three types of substitutions were generated: (1) alanine substitutions, (2) amino acid changes that maintained charge characteristics and (3) mutations that changed the charge at that position. After sequence confirmation of the substitutions, HCVpp infectivity of permissive Huh7 cells was assessed by infecting cells with HIV pseudotyped with wt or the RGE/RGD motif substitutions (Fig. 2). Infectivity was determined as a measure of luciferase activity and VSVG/HIV and EnvA/HIV were used as positive and negative controls, respectively.

The two amino acid substitutions generated at the first position, AGE and KGE, completely abrogated HCVpp infectivity, reducing luciferase levels down to 3 and 5% respective of wt. Changing the glycine to an alanine at the second position also reduced infectivity to 18%. As expected, changing RGE to RGD had no adverse affect on the viability of infection and resulted in 94% of wt. Interestingly, substituting the negatively charged aspartic acid (D) or glutamic acid (E) to a neutral alanine (A) did not impair infectivity of HCVpp, however a positive lysine (K) at this position reduced HCVpp to 14% of wt. This suggests that a negative or neutral charge at this position is critical in maintaining HCV E2 interactions with susceptible cells.

### Expression and incorporation of hepatitis C virus E1E2 RGE substitutions

**Figure 3:**
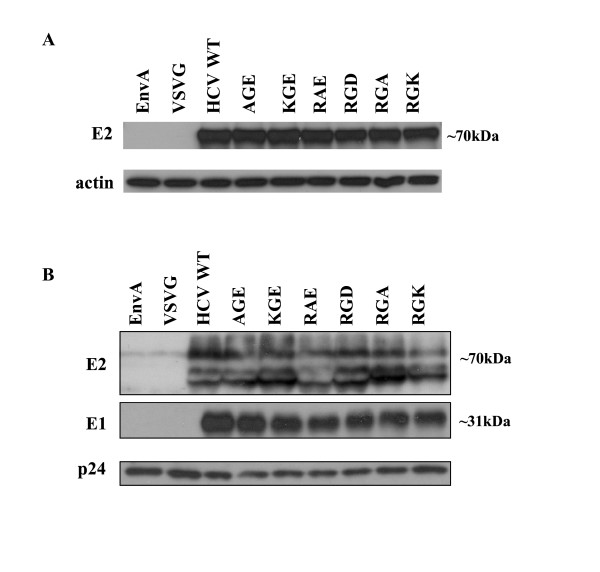
**Expression and incorporation of hepatitis C virus E1E2 glycoproteins in producer cell lysate and HCVpp**. (A) 293 T producer cells were lysed on analyzed by Western blot analysis using anti (α)-E2. Detection of actin was performed as a loading control. (B) Incorporation of HCV glycoproteins into HCVpp was determined by pelleting the virus through a 20% sucrose cushion followed by Western blot analysis. Detection of p24 capsid protein was performed as a loading control.

To confirm changes made in these amino acids did not adversely affect expression of the HCV E2 protein, expression levels in the 293 T producer cell lysates transiently transfected with HIV-luc backbone and the HCV E1E2 glycoprotein plasmids were examined by Western blot analysis. Blots were also probed for actin as a protein loading control. Cell lysates probed with anti-E2 antibody revealed a band of ~70 kDa for all wt and mutant glycoprotein transfected cells, corresponding to the size of the HCV E2 glycoprotein (Fig. 3A). All substitutions exhibited similar expression levels within producer cell lysate.

To determine if reduced infectivity of HCVpp could be attributed to less glycoprotein incorporation on the pseudoparticle, virions were pelleted through a 20% sucrose cushion and examined via Western blot for E2 incorporation. Protein p24 capsid of HIV was used as a loading control (Fig. 3B). E2 incorporation levels were similar for wt and for all substitutions generated within the RGE/RGD motif of E2. These results suggest that these substitution mutations of the RGE motif in E2 did not adversely affect E2 expression or incorporation.

### Inhibition of hepatitis C virus pseudoparticle entry using integrin ligands

**Figure 4:**
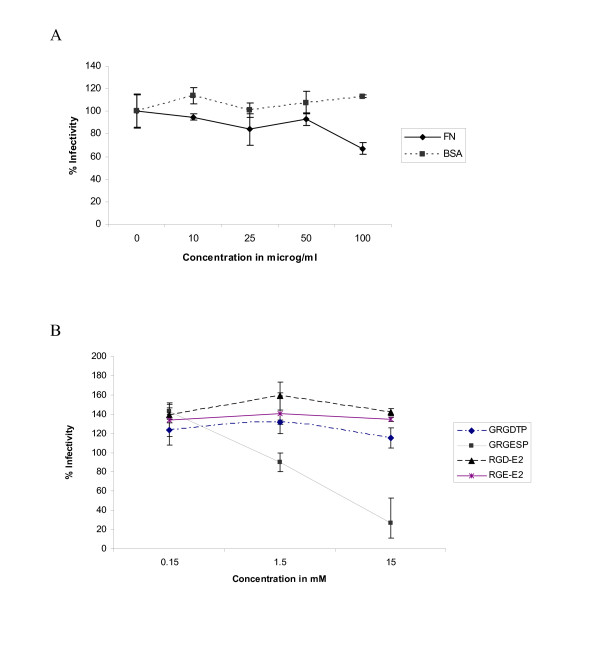
**Blocking RGE/RGD sites on Huh7 cells prior to hepatitis C virus pseudoparticle challenge**. (A) Huh7 cells were incubated at 4°C for 1 h with ECM proteins then washed and infected with HCVpp. (B) Inhibition of HCVpp infectivity by peptides GRGDTP, GRGESP, RGD-E2 or RGE-E2 was assessed by incubating Huh7 cells with increasing amounts of peptide at 4°C for 1 h, washing and infecting with HCVpp. Infecitivity for both was determined 72 h PI using a luciferase reporter assay.

To determine the potential role of integrins in HCV entry, Huh7 cells were incubated with extracellular matrix proteins before addition of HCVpp. Fibronectin (Fn), which is known to interact with integrins in an RGD dependent and independent fashion [33] and BSA were preincubated with Huh7 cells for 1 h at 4°C and then washed and infected with HCVpp. Seventy-two h PI luciferase reading were taken as a measure of entry. HCVpp entry was reduced to ~65–70% of wt at a Fn concentration of 100 μg/ml (Fig. 4A). BSA had no effect on HCVpp entry.

### Peptides with RGE motif inhibit hepatitis C virus pseudoparticle containing RGE motif

As genotype 1a strain H77 contains an RGE sequence, synthetic peptides with or without RGE or RGD sequences were incubated with Huh7 cells for 1 h at 4°C prior to HCVpp infection. Four synthetic peptides were used: GRGDTP, GRGESP and a RGD or RGE peptide followed by 15 amino acids corresponding to the sequence of the H77 E2 sequence (RGD-E2 and RGE-E2 respectively). The short, six amino acid peptide GRGESP inhibited HCVpp entry approximately 80% at a concentration of 15 mM, however the RGE-E2 peptide did not inhibit HCVpp (Fig. 4B). This could be due to the larger peptides folding on themselves, masking the RGE sequence. Inhibition by the GRGESP peptide suggests a potential RGE-dependent interaction with the Huh7 cell, therefore blocking HCVpp with integrin antibodies was pursued.

### Alpha integrin antibodies to do not block hepatitis C virus pseudoparticle entry

Seven monoclonal antibodies that functionally block the alpha subunit of integrins were incubated with Huh7 cells at 4°C for 1 h prior to infection with HCVpp. Antibodies directed against alpha subunits 1–6 and V were tested at a concentration of 20 μg/ml (data not shown). None of the antibodies used had an inhibitory effect on HCVpp infectivity, suggesting these alpha subunits are unlikely involved in the entry process of HCV.

### Characterization of RGE/RGD substitutions in CD81 binding

**Figure 5:**
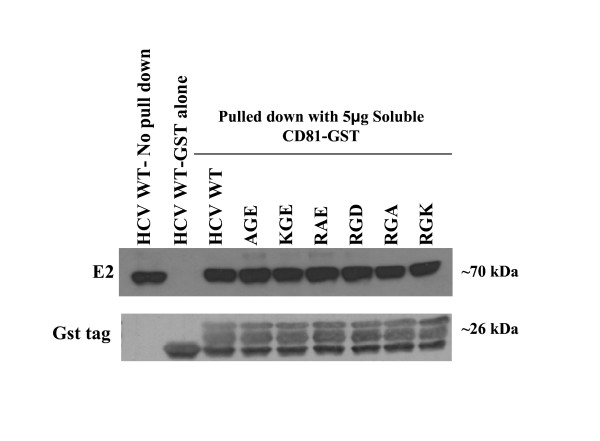
**Binding of hepatitis C virus E2 glycoproteins containing RGE substitutions to soluble CD81**. 293 T cells transfected with HCV E1E2 wt of mutant expression vectors were lysed 24 h post-transfection. Cleared cell lysate was incubated with soluble CD81-GST fusion protein. Binding to CD81 was determined by Western blot analysis of E2 and the GST tag. As a negative control, GST protein without soluble CD81 was incubated with HCV wt.

To investigate whether amino acid substitutions within the RGE/RGD domain of E2 reduced HCVpp infectivity through a disruption of CD81 binding, HCV E2 binding to soluble CD81 was assayed. Binding of HCV E1E2 proteins to a purified GST tag was used as a control. None of the amino acid substitutions interfered with CD81 and HCV E2 glycoprotein binding (Fig. 5). These results indicate that decrease in HCVpp infectivity is not due to a loss of CD81/E2 interaction.

### Conformation of RGE/RGD substitutions

**Figure 6:**
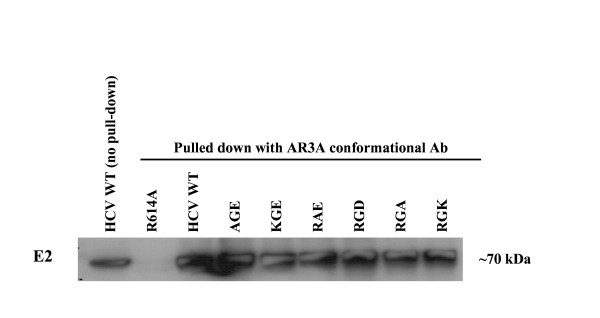
**Conformation of HCV E2 glycoprotein substitutions within conserved RGE/RGD motif**. 293 T cells transfected with HCV E1E2 wt or RGE/RGD mutant expression vectors were lysed 24 h post-transfection. Cleared cell lysate was incubated with AR3A (C1) conformational antibody to assess conformation of mutations. Immunoprecipitated proteins were detected by subsequent Western Blot analysis of E2. HCV E2 R614A was used as a negative control.

To confirm that loss of HCVpp infectivity was not due to a more general disruption of E2 structure in this region of the protein, we performed immunoprecipitation with an antibody that recognizes a conformational epitope within the putative CD81 binding regions 2 and 3 [34]. Wt and the amino acid substitutions as well as HCV E2 R614A, which was not detectable in a previous study and serves as a negative control [35], were analyzed in the immunoprecipitation. Except for the R614A negative control, all amino acid substitutions were captured with the conformational antibody, consistent with proper folding (Fig. 6).

## Discussion

In this study, we characterized an RGE/RGD motif at amino acids 648–650 of HCV E2. As integrins are frequently used as receptors for various viral pathogens, and many integrins recognize RGD sequences, this highly conserved RGE or RGD motif in the HCV glycoprotein E2 (see Fig. 1), led us to hypothesize that integrins might play a role in HCV binding and entry. While this three amino acid motif is suggestive of an integrin binding ligand, our data does not support this hypothesis. Rather, the large size of this conserved region is more likely indicative of a structural or other functional role for this region of the E2 glycoprotein.

Of the six amino acid substitutions introduced in this motif, only substitutions of the last position (aa 650) maintained infectivity when replaced with either an amino acid of the same, negative charge (substituting a glutamic acid with an aspartic acid) or with a neutral alanine. The E2 gene used in this study, derived from an H77 strain, contains an RGE at this position. Changing the glutamic acid to an aspartic acid, which is the other variation of this conserved motif, maintains infectivity levels 94% of RGE. A change in charge at this position to a positive amino acid (lysine) however, impaired infectivity significantly; dropping infection levels to only 14% of wt (see Fig. 2). Changes in the first two positions of this motif reduced HCVpp entry to 3–18%. Producer cell expression and viral particle incorporation of all of these substitutions were comparable to wt and could not account for the reduced HCVpp infectivity (see Fig. 3). Mutational analysis of this highly conserved, three amino acid motif underlines its importance in maintaining HCVpp infectivity.

Receptor binding sites of E2 are not fully understood; therefore we sought to determine whether or not this conserved region is involved in binding to CD81. We assayed the soluble-CD81 binding properties of RGE/D amino acid substitutions. Mutant E2 protein was expressed in 293 T cells and then assayed for its binding of soluble CD81 bound to a GST tag. A GST protein alone was utilized as a negative control. All mutants bound CD81 at wt level, suggesting that whatever role this region plays in the HCV replication cycle, it does not appear to relate to CD81 binding (see Fig. 5). Based on the conformational AR3A antibody analysis (see Fig. 6) and soluble CD81 binding (see Fig. 5), we can conclude that the CD81 binding epitopes are intact.

The conserved RGE/D initially led us to speculate that integrins were involved in mediating viral entry of HCV. Integrins have been shown to mediate binding of several viruses, including adenovirus, echovirus and hantavirus [36]. There are 16 α subunits and 8 β subunits that can interact in numerous ways to generate receptors of differing ligand specificity. Approximately 24 different heterodimers of αβ subunits have been identified, suggesting not all subunits are capable of interacting with one another [37]. We preincubated Huh7 cells with 1–6 and V alpha integrin blocking antibodies prior to HCVpp challenge to asses if infectivity was impaired. The inability of these blocking antibodies to prevent HCVpp infectivity (data not shown) suggests that at least these alpha subunits, are not involved in HCV entry. This conclusion is consistent with the other results in this study, in particular the mutational analysis that demonstrates that RGA maintains HCVpp entry.

Peptides containing either an RGD or RGE motif were preincubated with Huh7 cells to determine if masking a potential ligand site on the cell surface could impact HCVpp infectivity. Of the four peptides generated, the short, six amino acid peptide GRGESP, reduced HCVpp infectivity by approximately 80% at 15 mM (see Fig. 4B). If integrins were directly involved, we would expect to see the short RGD containing peptide having the same inhibitory effect on HCVpp entry, however it did not impair HCVpp entry. The other RGE containing peptide, RGE-E2, which is followed by 15 amino acids corresponding to the HCV E2 H77 strain, did not affect HCVpp entry. This could be due to the peptide folding on itself and masking the RGE sequence. Furthermore, addition of fibronectin prior to HCVpp challenge did not have a significant effect on inhibiting HCVpp entry (see Fig. 4A) suggesting that the function of this region is not RGE/D dependent binding to integrins.

As sequence alignment demonstrates a much larger span of conservation than simply these three residues (see Fig. 1), we suspect this entire stretch of conserved amino acids is critical to HCV infection. It is possible that this conserved region, including the RGE/RGD motif of E2, is critical for the proper folding and structure of E2. Just downstream of this three amino acid motif is the transmembrane domain, which has been well defined in its involvement in entry [38-41]. Further investigation is needed to dissect the exact role of this highly conserved region of E2 in HCV entry, however this work clearly demonstrates it plays a key role in the HCV replication cycle.

## Methods

### Cell lines and antibodies

293 T human embryonic kidney cells were maintained in Dulbecco's modified Eagle's media (DMEM) supplemented with 10% fetal calf serum with penicillin, streptomycin. Huh7 and Hep3B cells were maintained in DMEM supplemented with 10% fetal calf serum, penicillin, streptomycin and supplemented with 5 ml Hepes (1 M) (Gibco), and Nonessential amino acids (NEAA) (Gibco). The goat polyclonal antibody against hepatitis C virus (HCV) E2 and the monoclonal mouse antibody for E1 glycoproteins (GP) (genotype 1a) were obtained through ViroStat. The mouse anti-HIV p24 monoclonal antibody was obtained from the National Institutes of Health AIDS Research and Reference Reagent Program. Polyclonal rabbit glutathione-S-transferase (GST) antibody was obtained from NeoMarkers. The conformational anti-E2 AR3A antibody was provided by Dennis Burton, PhD from The Scripps Research Institute.

### Mutagenesis of the HCV E2 glycoprotein gene

The cDNA clone containing E1E2 from genotype 1a strain H77 in pCB6, was kindly provided by Charles Rice, PhD (Rockefeller University). All alanine substitution mutations of the HCV E2 glycoprotein were generated by site-directed mutagenesis with the Stratagene Quick-Change mutagenesis kit according to the supplier's protocols. All mutations were confirmed by DNA sequencing.

### Pseudotyping

Pseudotyped viruses were produced by cotransfecting DNA encoding wild-type (wt) or mutant glycoproteins with the Env-deficient HIV vector carrying a luciferase reporter gene (pNL4-3-Luc-R^-^-E^-^) into 293 T producer cells. One microgram of the wt or mutant glycoprotein expression plasmid and 3 μg of pNL4-3-Luc-R^-^-E^- ^were used to transfect 293 T cells (90% confluent) in 6-well plates with polyethylenimine (PEI). The DNA cocktail was added to 200 μl Opti-MEM (Gibco) media and PEI was added at 2× the volume of DNA. The mixture was incubated at room temperature for 15 min. 293 T producer cells were rinsed with PBS (no Ca^++^/no Mg^++^). Eight hundred microliters of Opti-MEM (Gibco) was added to each well and the PEI/DNA mixture was added. After 5–6 h incubation at 37°C, the DNA cocktail was aspirated off and 3 ml cell culture media was added per well. A minimum of two wells per mutant were done at each time, for a total of 6 ml. The supernatants containing the pseudotyped viruses were collected 48 h post-transfection (PT) and filtered through a 0.45 μm-pore-size filter (Nalgene).

### Pseudotyped virus infectivity assay

Huh7 cells were seeded in 12-well plates at a density of 8 × 10^4 ^per well one day prior to infection. Cells were incubated with 500 μl of pseudotyped virus for 6 h, then virus was removed and cell growth media was added. The cells were lysed in 200 μl of cell culture lysis reagent (Promega) at 72 h post-infection (PI). The luciferase activity was measured with a luciferase assay kit (Promega) and a FB12 luminometer (Berthold detection system) according to supplier's protocol. Each sample was done in duplicate and experiments were repeated at least three times.

### Western blot analysis

To determine HCV E1E2 expression and incorporation, 293 T producer cells transfected with HCV E1E2/HIV plasmids as described above, were lysed in 0.5 ml of 1% Triton X-100 lysis buffer (50 mM Tris-HCl [pH 7.5], 150 mM NaCl, 5 mM EDTA) and protease inhibitor cocktail (10 μg/ml leupeptin and pepstatin, 5 μg/ml aprotinin and 2 mM phenylmethylsulfonyl fluoride) after harvesting virus and rinsing cells with PBS (no Ca^++^/no Mg^++^). The protein samples were spun down at 14 k for 10 min to clear cellular debris and transferred to fresh eppendorf tubes. SDS-PAGE loading dye was added to the protein samples, which were subsequently boiled for 5 min at 95°C, followed by sodium dodecyl sulfate-polyacrylamide gel electrophoresis (SDS-PAGE) and transferred to a polyvinyl difluoride membrane (PVDF). Membranes were then probed for HCV glycoproteins E1 and E2 and actin, p24 or GST using peroxidase-conjugated secondary antibody and chemiluminescence reagent according to the supplier's protocol (SuperSignal West pico chemiluminescent substrate, Pierce). To determine incorporation of E1 and E2 into the pseudotyped viruses, 4 ml of pseudotyped virus was layered onto a 1 ml cushion of 20% sucrose in PBS and centrifuged at 55,000 rpm for 45 min in a SW55Ti rotor (Beckman Coulter) at 16°C. The pelleted pseudovirions were lysed in 50 μl of 1% Triton X-100 lysis buffer and subjected to SDS-PAGE and Western blot analysis.

### Bovine serum albumin and fibronectin blocking assay

Huh7 cells were seeded in 12-well plates at a density of 8 × 10^4 ^per well one day prior to infection. Growth media was aspirated off and Fibronectin (GibcoBRL) or Bovine Serum Albumin (BSA) (Fisher) was added in DMEM to cells. Cells were incubated at 4°C for 1 h. Unbound BSA or Fibronectin was rinsed off with cold DMEM. Cells were incubated with 500 μl of pseudotyped virus for 6 h, then virus was removed and cell growth media was added. The cells were lysed in 200 μl of cell culture lysis reagent (Promega) at 72 h post-infection (PI). The luciferase activity was measured with a luciferase assay kit (Promega) and a FB12 luminometer (Berthold detection system) according to supplier's protocol. Each sample was done in duplicate and repeated twice.

### Custom peptide blocking assay

Custom peptides generated were as follows: GRGDTP, GRDESP, RGDRCDLEDRDRSELSPL (RGD-E2) and RGERCDLEDRDRSELSPL (RGE-E2). Huh7 cells were seeded in 12-well plates at a density of 8 × 10^4 ^per well one day prior to infection. Growth media was aspirated off and peptides were added in DMEM to cells. Cells were incubated with peptides at 4°C for 1 h. Unbound peptides were rinsed off with cold DMEM. Cells were incubated with 500 μl of pseudotyped virus for 6 h, then virus was removed and cell growth media was added. The cells were lysed in 200 μl of cell culture lysis reagent (Promega) at 72 h post-infection (PI). The luciferase activity was measured with a luciferase assay kit (Promega) and a FB12 luminometer (Berthold detection system) according to supplier's protocol. Each sample was done in duplicate and repeated twice.

### Alpha integrin blocking assay

Alpha integrin blocking antibody kit was purchased through Millipore. This kit contained blocking antibodies for alpha subunits 1–6 and V. Huh7 cells were seeded in 12-well plates at a density of 8 × 10^4 ^per well one day prior to infection. Growth media was aspirated off and antibodies were added to cells along with HCVpp at a final concentration of 20 μg/ml. Cells were incubated with blocking antibodies and HCVpp at 37°C for 3 1/2 h. The virus and antibody cocktail was then removed and cell growth media was added. The cells were lysed in 200 μl of cell culture lysis reagent (Promega) at 72 h post-infection (PI). The luciferase activity was measured with a luciferase assay kit (Promega) and a FB12 luminometer (Berthold detection system) according to supplier's protocol. Each sample was done in triplicate.

### CD81 binding assay

The CD81 clone used was kindly provided by Shoshana Levy, PhD (Stanford University). A glutathione S-transferase (GST) fusion protein containing the large extracellular loop (LEL) of human CD81 was generated as previously described [5]. 293 T producer cells were transfected with 1 μg HCV E1E2 DNA using PEI. After 48 h cells were lysed in 0.5% Triton X-100 lysis buffer with protease inhibitor on ice for 30 min. Cell lysates were clarified by centrifuging at 20,000 × g for 30 min at 4°C. Two-hundred microliters of clarified lysates from these cells were incubated with 5 μg of CD81-GST fusion protein or GST protein alone with gentle rocking at 4°C for 16 h. Fifty microliters of Glutathione Sepharose 4B (GSH) beads (GE Healthcare) rinsed three times with PBS (140 mM NaCl, 27 mM KCl, 10 mM Na_2_HPO_4_, 1.8 mM KH_2_PO_4_) were added and incubated at 4°C for 1 h. The slurry was spun down for 1 min at 14, 000 rpm and GSH beads were rinsed two times with 0.5% Triton X-100 lysis buffer. SDS-PAGE loading dye was added to the beads and samples were boiled at 95°C for 5 min. Slurry was spun down again and supernatant was collected for SDS-PAGE and Western blot analysis.

### E2 conformational antibody immunoprecipitation

293 T producer cells were transfected with 1 μg wt or a selection of CD81 binding deficient or binding competent mutant HCV E1E2 DNA constructs using PEI. After 48 h cells were lysed in 0.5% Triton X-100 lysis buffer with protease inhibitor on ice for 30 min. Cell lysates were clarified by centrifuging at 20,000 × g for 30 min at 4°C. Four-hundred microliters of clarified lysates from these cells were incubated with 1 μg of AR3A conformational antibody [34] with gentle rocking at 4°C for 16 h. Immobilized protein A (Pierce) beads were rinsed three times with PBS (140 mM NaCl, 27 mM KCl, 10 mM Na_2_HPO_4_, 1.8 mM KH_2_PO_4_). Fifty microliters of rinsed polyA beads were then added to the cell lysate/antibody cocktail and incubated with gentle rocking at 4°C for 2 h. Beads were washed three times with 100 μl 0.5% Triton lysis buffer. SDS-PAGE loading dye was added to the beads and samples were boiled at 95°C for 5 min. Slurry was spun down and supernatant was collected for SDS-PAGE separation and Western blot analysis with a polyclonal anti E2 antibody (Virostat).

## Competing interests

The authors declare that they have no competing interests.

## Authors' contributions

KBR participated in the design of the study, performed the experiments and drafted the manuscript. LR designed the study and participated in drafting the manuscript.
